# List of Physicians Who Have Died of Yellow Fever in Memphis

**Published:** 1878-12

**Authors:** 


List of Physicians who have died of Yellow Fever in Memphis.—Dr. R. W. Mitchell, Medical Director, Howard Corps, Memphis, Tennessee, has furnished the following list of physicians who have died in Memphis since the commencement of the present epidemic up to Oct. 1, 1878:—Armstrong, J. W., Memphis, Tennessee; Burcham, Robert, Columbus, Ohio; Bond, T. W., Brownsville, Tenn.; Bankson, T. S., Stevenson, Alabama; Chevis, L. A., Savannah, Georgia; Dawson, G. R., Memphis, Tennessee; Forbes, J. G., Round Rock, Texas; Gorrell, J. O. G., Fort Wayne, Indiana; Harlan, L. B., Hot Springs, Arkansas; Hodges, W. R., Memphis, Tenn.; Hicks, Jno. B., Murfreesboro, Tenn.; Meade, W. C., Memphis, Tenn.; McKim, J. W., St. Louis, Missouri; McGregor, Thos. H., Tipton County, Tenn.; Menees, T. W., Nashville, Tenn.; Nugent, P. C., St. Louis, Missouri; Pierce, Hiram M., Cincinnati, Ohio; Renner, J. G., Indianapolis, Indiana; Turck, P., Cincinnati, Ohio; Williams, R. B., Woodburn, Kentucky; Robbins, N. H., Memphis, Tenn.; Earley, E. T., Little Rock, Arkansas; White, J. M. S., Macon, Georgia. The above were all members of the Howard Volunteer Medical Corps. Dr. B. W. Arent, Dr. Paul Otey, Dr. John H. Erskine, Dr. J. P. Watson, Dr. Jno. D. Woodward, resident physicians of Memphis.

Physicians who died while in attendance on yellow fever patients:—Dr. T. W. Meares, volunteer from Nashville, Tenn.; Dr. J. B. Hicks, volunteer from Murfreesboro, Tenn.; Dr. J. S. Bankson, volunteer from Stevenson, Alabama; Dr. R. H. Tate, volunteer from Cincinnati, Ohio; Dr. W. J. Armstrong of Memphis. Sept. 30th, Dr. Edward Tandy Easley, volunteer from Little Rock, Arkansas. Oct. 6th, Dr. Wm. R. Lowry, of Memphis; Dr. R. F. Brown, Secretary of Board of Health. Oct. 7th, Dr. J. F. Sample, volunteer from Austin, Miss.

*Vicksburg, Miss.*—Oct, 6th, Dr. Newman. Oct. 7th, Dr. M. Blackburn, Dr. Birdsong.

*Tangipahoa, La.*—Sept. 18th, Dr. H. A. Swasey.

*Grand Junction, Tenn.*—Oct. 1st, Dr. Joseph H. Prewitt.

*Greenville, Miss.*—Dr. Stafford, Dr. V. F. P. Alexander.

*Port Gibson, Miss.*—Oct. 1st, Dr. J. G. Strobridge, Dr. W. D. Spratt.

Previously reported, 63. Total to date, 81.—*Medical Journal.*

